# Royal Jelly Supplementation Improves Menopausal Symptoms Such as Backache, Low Back Pain, and Anxiety in Postmenopausal Japanese Women

**DOI:** 10.1155/2018/4868412

**Published:** 2018-04-29

**Authors:** Takashi Asama, Hidenori Matsuzaki, Shinobu Fukushima, Tomoki Tatefuji, Ken Hashimoto, Takashi Takeda

**Affiliations:** ^1^Institute for Bee Products and Health Science, Yamada Bee Company, Inc., Okayama, Japan; ^2^Division of Women's Health, Research Institute of Traditional Asian Medicine, Kindai University, Osaka, Japan

## Abstract

**Objectives:**

This study aimed to evaluate the effect of Royal Jelly (RJ) at a dose of 800 mg/day on menopausal symptoms in healthy Japanese postmenopausal women with placebo-controlled design.

**Material and Methods:**

A total of 42 healthy Japanese postmenopausal women have been recruited for this study. The subjects were randomized to oral treatment with either 800 mg of protease-digested lyophilized powder of RJ (enzyme-treated RJ) or placebo (800 mg of dextrin) daily for 12 weeks. The level of menopausal symptoms has been evaluated every 4 weeks, using menopausal symptoms questionnaire of Japanese women. Independent *t*-test was used to evaluate statistical significance of the treatment effects between the two groups.

**Results and Conclusion:**

All of the 42 women have completed the trial. There were significant differences related to the anxiety score (*P* = 0.046) and backache and low back pain score (*P* = 0.040) between 800 mg/day enzyme-treated RJ and placebo-treated groups after 12 weeks of administration, and no significant differences were found between the two groups in 4 weeks after intervention. No side effects were observed in either group. This study demonstrates that enzyme-treated RJ supplementation with doses of 800 mg/day is effective in relieving menopausal symptoms such as anxiety, backache, and low back pain in Japanese postmenopausal women.

## 1. Introduction

 Menopause is a normal part of women's life, typically between ages 45 and 55 years, when the ovaries naturally stop producing eggs and their menstrual periods end. The decreased production of estrogen level affects autonomic nervous system, which can directly trigger menopause symptoms such as hot flashes, insomnia, mood changes, fatigability, neck stiffness, backache, and low back pain and headache. Particularly, neck stiffness, headache, and low back pain are the main menopausal symptoms in Japanese women [1]. Hormone replacement therapy (HRT) has been widely accepted to relieve menopausal symptoms [2, 3], while it is associated with an increased risk of breast cancer [3, 4]. Therefore, an effective and safe alternative health product for women is needed to improve menopausal symptoms. Various safe alternative health products such as isoflavones, St John's wort, and Royal Jelly (RJ) can provide relief of menopausal symptoms [5–7].

Among them, RJ has been long used as a nutritional supplement and its safety has been demonstrated by several clinical studies [8–10], so RJ supplementation could be a good option for those having concerns about HRT. Traditionally, RJ has been used to improve menopausal symptoms [11], and case reports have revealed that the menopausal symptoms such as hot flashes, anxiety, irritation, fatigability, neck stiffness, low back pain, and headache were improved with RJ supplementation in menopausal women [7]. However, the placebo-controlled studies of RJ on menopausal symptoms have not been reported. RJ contains a considerable amount of proteins, free amino acids, lipids, vitamins, minerals, and unique fatty acids such as* (E)*-10-hydroxy-2-decenoic acid (10HDA) and 10-hydroxydecanoic acid (10HDAA) [12]. It is reported that RJ and 10HDA have estrogenic effect and agonist activity for estrogen receptor (ER) *β* [13, 14]; hence one of the mechanisms for improvement of physical and psychiatric symptoms in menopause is considered to be the estrogenic effect.

Here, a randomized double-blind placebo-controlled study was conducted in order to determine the effect of RJ on menopausal symptoms, specifically in postmenopausal women.

## 2. Material and Methods

### 2.1. Participants

The study was carried out in accordance with the principles outlined in the Declaration of Helsinki. Written informed consent was obtained from all the participants before enrollment. The study protocol was approved by Shiba Palace Clinic Ethics Committee (Tokyo, Japan). This study has been conducted between July 2015 and December 2015 (UMIN000018842). Study volunteers have been recruited from volunteer bank of Research Center for Immunological Analysis, Inc. (Okayama, Japan) and CROèe Inc. (Tokyo, Japan). All participants were healthy Japanese postmenopausal women (at least 12 month have passed since their last menstrual period) with estrogen level more stable than premenopausal women. Major eligibility criteria were having undergone natural menopause within 5 years, having self-rating depression scale score < 49, having hot flash score > 0, and being diagnosed as having menopausal symptoms which require no treatment by obstetrician. Major exclusion criteria were having undergone treatment with sexual hormones, nonhormonal climacteric drugs, or any treatment to alleviate menopausal symptoms, having undergone treatment with chemical or plant-derived medicines, having severe diseases (e.g., of heart, liver, kidneys, digestive system, or metabolic diseases), or having undergone intake of drugs and supplements which could influence the outcome of this study.

### 2.2. Study Design

This study has been conducted as a double-blind randomized controlled trial. Forty-two women were randomized into the placebo group and enzyme-treated RJ (YRP-M-141122) group by randomly distributing numerical codes. After randomization, the codes were kept in the possession of one employee of Yamada Bee Company, Inc., who was not involved in the present study until completion. It can be noted that sample size calculation was not estimated because the preliminary study was not conducted. The enzyme-treated RJ powder was obtained from Yamada Bee Company, Inc. (Okayama, Japan). It was standardized to include minimum 3.5% 10HDA and minimum 0.6% 10HDAA and provided as tablets. Each tablet contained 200 mg of enzyme-treated RJ. The enzyme-treated RJ group received four tablets once a day (800 mg/day) for 12 weeks. For placebo preparations, dextrin was used instead of enzyme-treated RJ. The placebo tablets were also administered once a day for 12 weeks. Additionally, post intervention follow-up was conducted for 4 weeks. During this follow-up, all study participants had not ingested enzyme-treated RJ or placebo.

### 2.3. HPLC Analysis

**Figure 1:**
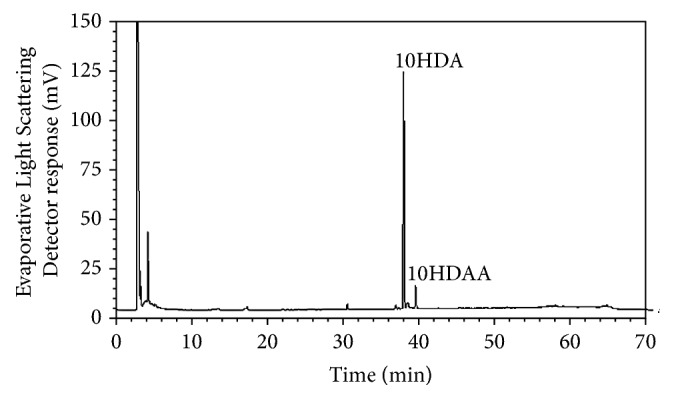
The HPLC chemical fingerprint for the extract of enzyme-treated RJ powder.

10HDA and 10HDAA in the RJ powder were analyzed as follows. RJ tablets were crushed in a mortar and extracted with methanol under agitation at room temperature. The concentration of 10HDA and 10HDAA in the extract was measured using an HPLC system (Shimadzu, Kyoto, Japan) equipped with a Cosmosil 5C18-AR-II column 4.6 mm ID × 250 mm (Nacalai Tesque, Kyoto, Japan) at 40°C under a constant flow rate (1.0 mL/min) of 25% acetonitrile in 0.1% TFA (mobile phase). Detection was performed with PDA detector at 215 nm for 10HDA and evaporative light scattering detector (ELSD, 30°C) for 10HDAA. The HPLC chemical fingerprint for the extract can be seen in Figure 1, which was recorded by ELSD (50°C), with the gradient solvent system of methanol {5% (5 min hold), 5–90% (45 min linear gradient), 100% (10 min hold), and 5% (10 min hold)} in 0.1% TFA at a flow rate of 1.0 mL/min.

### 2.4. Evaluation of Menopausal Symptoms

**Figure 2:**
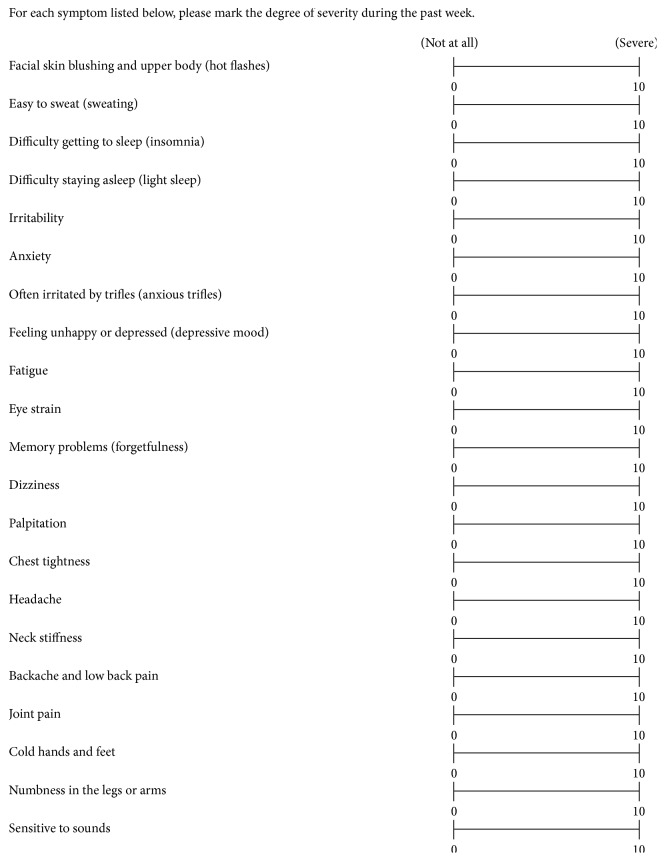
Menopausal symptoms questionnaire of Japanese women.

Menopausal symptoms questionnaire of Japanese women [15] (Figure 2) was used to assess menopausal symptoms. This assessment tool of menopausal symptoms is based on common complaints in Japanese women which include facial skin blushing and upper body (hot flashes), easy to sweat (sweating), difficulty getting to sleep (insomnia), difficulty staying asleep (light sleep), irritability, anxiety, often irritated by trifles (anxious trifles), feeling unhappy or depressed (depressive mood), fatigue, eye strain, memory problems (forgetfulness), dizziness, palpitations, chest tightness, headache, neck stiffness, backache and low back pain, joint pain, cold hands and feet, numbness in the legs or arms, and sensitive to sounds. Symptoms like irritation, dizziness, backache and low back pain, and joint pain had high priority for investigation based on the result of our previous studies which have shown an improvement with RJ treatment [16]. Other menopausal symptoms were the secondary outcome. The Visual Analog Scale (VAS) was used to assess each above-described menopausal symptom. The participants answered menopausal symptoms questionnaire of Japanese women during the week before randomization and after 4, 8, and 12 weeks of daily intake and 4 weeks after intervention.

### 2.5. Statistical Analysis

Statistical analysis was performed with JMP software (version 5.1, SAS Institute). The independent *t*-test was used to compare the changes from baseline to those after intervention between the two groups. *P* < 0.05 was considered significant for all data analyses.

## 3. Results

### 3.1. Participants

**Figure 3:**
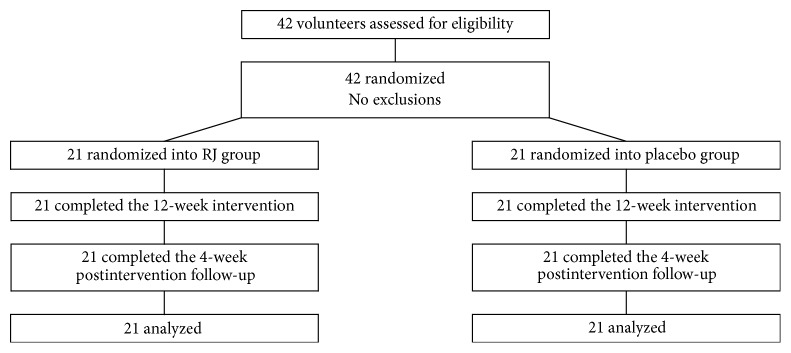
Trial flow diagram.

All of the 42 women (21 randomized into placebo-treated group and 21 randomized into the enzyme-treated RJ group) completed the trial (Figure 3). The baseline characteristics like age, body mass index (BMI), postmenopausal periods, and each menopausal symptom score were not significantly different between the two groups (Table 1). During the study, no side effects associated with enzyme-treated RJ intake were observed.

### 3.2. Effects of Enzyme-Treated RJ Treatment on Menopausal Symptoms

**Figure 4:**
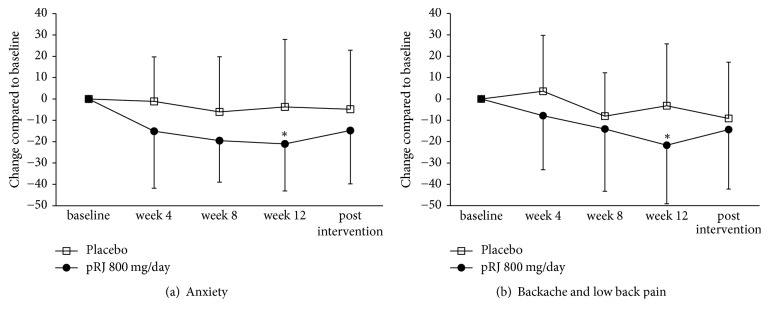
Enzyme-treated RJ treatment improves the score of menopausal symptoms such as backache and low back pain and anxiety. A *P* value of <0.05 was considered statistically significant compared to placebo and is indicated by the asterisk. All values are mean ± SD.

There were significant differences in the anxiety score (*P* = 0.046) and backache and low back pain score (*P* = 0.040) between 800 mg/day enzyme-treated RJ and placebo-treated groups after 12 weeks of administration, and no significant differences were found between the two groups after intervention (Figure 4 and Table 2). Regarding hot flashes, there were significant differences between the two groups after 8 weeks of administration and no significant differences after 12 weeks of administration (Table 2). No significant change was observed in any other menopausal symptom score between the two groups (Table 2).

## 4. Discussion

The present study showed that enzyme-treated RJ supplementation with doses of 800 mg/day has improved the anxiety score and backache and low back pain score in healthy Japanese postmenopausal women. Hot flashes are the most common symptom of menopause in Western countries [17]. However, Melby et al. showed that the main menopausal symptoms in Japanese women are not hot flashes but neck stiffness, headache, and low back pain [1], so the relief of backache and low back pain confirmed in the present study is considered to be meaningful in Japanese women.

The beneficial effects of RJ on menopausal symptoms were reported only in controlled before-after studies [7], so the present study is the first double-blind randomized placebo-controlled trial to investigate the effect of enzyme-treated RJ on menopausal symptoms. The case report of Kushima et al. showed that RJ intake for 10–14 days of 500 or 1,000 mg/day dose improved anxiety and backache and low back pain [7], and our previous study suggested that enzyme-treated RJ intake for 8 weeks of 800 mg/day dose improved psychological symptoms and backache and low back pain compared with those before intake [16]. These findings support the result of the present study.

Although it is not clear exactly how enzyme-treated RJ improves menopausal symptoms, we presume that its estrogenic activity could play an important role for selective ER modulators [13, 14]. Krezel et al. described that an anxiety behavior increased in ER*β* -deficient female mice [18], and Imwalle et al. showed that ER*β*-deficient female mice had significantly lower serotonin content in several brain regions (the bed nucleus of the stria terminalis, preoptic area, and hippocampus) [19]. The 10HDA and 10HDAA content of RJ showed ER*β*-selective agonist activity [13], and Ito et al. suggested that the intraperitoneal injection of 10HDA has been effective in protecting against anxiety in stress-inducible depression-like mouse model [20]. In light of these reports, ER*β* agonist activity of 10HDA and 10HDAA could be one contributor in reducing anxiety through an increase in serotonin. The serotonin has long been considered to have an important role in the control of pain [21], so the one mechanism for amelioration of low back pain may be the increase in serotonin. Additionally hypoperfusion is also believed to be other cause of low back pain [22, 23]. Our unpublished data had shown that 10HDAA treatment increased blood flow in rats; thus the improvement of blood flow due to 10HDAA may contribute to improving low back pain.

10HDA and 10HDAA have effect on ER*β* as described above [13] but have little effect on ER*α* [13] which is a risk factor for cancer [24, 25], so RJ is considered to be a safer alternative health product.

This study has some limitations which have to be pointed out: (1) there was a reduced number of participants (21 postmenopausal women per group), (2) lower effective dose and dose dependency were unclear, (3) the subjects were only postmenopausal women, and (4) the detailed mechanism and component for improvement of menopausal symptoms were not characterized. Further studies are needed to investigate the above issues.

## 5. Conclusion

This study demonstrates that enzyme-treated RJ supplementation with doses of 800 mg/day has beneficial effects on menopausal symptoms such as anxiety, backache, and low back pain in Japanese postmenopausal women.

## Figures and Tables

**Table 1:** Baseline characteristics of the study population.

	Placebo	RJ	*P*
Subject number	21	21	N. D
Postmenopausal period			
1–3 years	16	15	
3–5 years	5	6	
Age (years)	54.4 ± 3.0	54.4 ± 2.6	1.000
BMI (kg/m^2^)	21.2 ± 2.6	21.0 ± 3.0	0.790
Each menopausal VAS score			
Hot flashes	41.5 ± 35.0	44.2 ± 32.9	0.797
Sweating	44.6 ± 30.2	52.0 ± 30.1	0.436
Insomnia	27.3 ± 27.4	30.7 ± 26.1	0.684
Light sleep	42.0 ± 28.5	43.2 ± 23.0	0.873
Irritability	32.5 ± 33.0	34.0 ± 25.0	0.871
Anxiety	28.7 ± 26.6	30.5 ± 22.5	0.818
Anxious trifles	35.1 ± 31.1	35.5 ± 25.8	0.970
Depressive mood	30.2 ± 24.3	33.4 ± 26.3	0.685
Fatigue	39.0 ± 23.7	42.5 ± 22.0	0.620
Eye strain	60.7 ± 31.7	58.6 ± 27.8	0.821
Forgetfulness	58.3 ± 28.2	57.7 ± 26.4	0.942
Dizziness	27.1 ± 29.0	27.4 ± 28.7	0.970
Palpitation	25.9 ± 27.9	18.4 ± 22.2	0.342
Chest tightness	17.2 ± 20.7	7.1 ± 10.1	0.051
Headache	46.9 ± 33.8	34.9 ± 29.4	0.229
Neck stiffness	65.4 ± 31.7	63.6 ± 31.6	0.854
Backache and low back pain	47.0 ± 34.7	47.2 ± 30.9	0.989
Joint pain	39.0 ± 33.3	37.5 ± 26.6	0.875
Cold hands and feet	38.4 ± 28.0	41.7 ± 30.4	0.717
Numbness in the legs or arms	25.1 ± 28.9	21.8 ± 26.5	0.703
Sensitive to sounds	31.5 ± 32.2	18.9 ± 20.5	0.137

RJ, enzyme-treated Royal Jelly. The values are means ± SD.

**Table 2:** Effect of enzyme-treated RJ treatment on menopausal symptoms.

	Treatment group	Change compared to baseline							
		Week 4	*P*	Week 8	*P*	Week 12	*P*	Week 16 (post intervention)	*P*
Hot flashes	Placebo	−4.5 ± 14.5	0.208	−12.5 ± 19.7	**0.026**	−16.8 ± 26.7	0.363	−19.0 ± 23.0	0.372
	RJ	−13.1 ± 27.2		−28.5 ± 25.0		−25.2 ± 32.1		−27.0 ± 33.2	
Sweating	Placebo	−7.5 ± 20.7	0.272	−18.8 ± 25.9	0.149	−22.0 ± 31.2	0.415	−26.6 ± 28.9	0.533
	RJ	−15.4 ± 24.8		−29.3 ± 20.1		−29.5 ± 27.5		−32.0 ± 26.4	
Insomnia	Placebo	−12.0 ± 23.2	0.788	−15.8 ± 22.3	0.529	−15.3 ± 24.7	0.961	−16.3 ± 28.6	0.694
	RJ	−9.8 ± 27.8		−11.0 ± 26.1		−14.9 ± 25.6		−13.1 ± 22.5	
Light sleep	Placebo	−6.9 ± 32.3	0.568	−24.1 ± 26.4	0.752	−20.8 ± 24.5	0.676	−21.0 ± 26.5	0.954
	RJ	−12.8 ± 34.2		−21.6 ± 25.9		−24.2 ± 27.4		−21.5 ± 31.4	
Irritability	Placebo	−2.3 ± 22.2	0.080	−12.4 ± 18.4	0.207	−8.9 ± 20.9	0.252	−9.0 ± 22.6	0.420
	RJ	−15.5 ± 25.2		−20.6 ± 22.7		−16.8 ± 23.4		−15.5 ± 28.5	
Anxiety	Placebo	−1.1 ± 20.9	0.067	−6.0 ± 25.8	0.063	−3.7 ± 31.6	**0.046**	−4.8 ± 27.7	0.229
	RJ	−15.1 ± 26.7		−19.5 ± 19.4		−21.0 ± 22.1		−14.8 ± 25.0	
Anxious trifles	Placebo	−6.7 ± 19.2	0.343	−11.0 ± 14.8	0.136	−10.5 ± 22.5	0.213	−13.7 ± 23.0	0.645
	RJ	−14.3 ± 30.6		−21.2 ± 26.7		−19.8 ± 24.7		−17.2 ± 26.0	
Depressive mood	Placebo	−3.5 ± 24.5	0.081	−9.9 ± 21.2	0.125	−6.0 ± 30.9	0.075	−7.2 ± 29.8	0.185
	RJ	−17.2 ± 25.2		−21.3 ± 25.8		−22.3 ± 26.9		−19.2 ± 28.0	
Fatigue	Placebo	−14.4 ± 28.7	0.576	−19.4 ± 30.9	0.502	−22.9 ± 25.0	0.284	−18.3 ± 26.2	0.475
	RJ	−19.0 ± 23.7		−24.9 ± 20.5		−31.0 ± 23.5		−23.6 ± 21.0	
Eye strain	Placebo	−4.3 ± 23.3	0.697	−14.4 ± 21.8	0.655	−14.6 ± 26.5	0.885	−11.5 ± 25.3	0.778
	RJ	−7.5 ± 29.2		−18.5 ± 35.6		−16.0 ± 34.5		−8.9 ± 32.9	
Forgetfulness	Placebo	−7.0 ± 22.4	0.435	−9.9 ± 22.9	0.156	−14.1 ± 27.6	0.373	−19.2 ± 29.0	0.750
	RJ	−12.3 ± 21.8		−21.2 ± 27.5		−21.7 ± 26.9		−16.1 ± 33.3	
Dizziness	Placebo	−7.8 ± 17.9	0.169	−5.0 ± 18.5	0.091	−11.7 ± 19.9	0.333	−15.1 ± 20.7	0.884
	RJ	−18.3 ± 29.6		−18.2 ± 29.5		−19.8 ± 32.2		−15.9 ± 31.0	
Palpitation	Placebo	−3.4 ± 17.7	0.978	−7.2 ± 21.5	0.229	−14.1 ± 20.0	0.152	−9.4 ± 19.5	0.587
	RJ	−3.6 ± 16.3		0.9 ± 21.5		−5.8 ± 16.9		−6.1 ± 19.4	
Chest tightness	Placebo	−5.0 ± 16.5	0.522	−4.6 ± 19.9	0.312	−6.0 ± 17.9	0.257	−8.4 ± 17.2	0.056
	RJ	−2.2 ± 10.5		1.5 ± 18.7		−0.3 ± 14.1		1.3 ± 14.6	
Headache	Placebo	−3.9 ± 19.2	0.499	−8.2 ± 23.7	0.819	−7.5 ± 29.0	0.602	−13.5 ± 28.6	0.673
	RJ	−8.6 ± 25.1		−6.3 ± 30.8		−12.0 ± 26.1		−10.1 ± 22.6	
Neck stiffness	Placebo	−4.0 ± 14.5	0.708	−10.7 ± 19.1	0.231	−14.0 ± 22.5	0.805	−14.2 ± 20.6	0.971
	RJ	−6.0 ± 20.9		−17.4 ± 16.6		−15.7 ± 22.2		−14.5 ± 21.4	
Backache and low back pain	Placebo	3.6 ± 26.2	0.156	−8.0 ± 20.3	0.440	−3.2 ± 29.0	**0.040**	−9.1 ± 26.4	0.535
	RJ	−7.9 ± 25.3		−14.1 ± 29.2		−21.7 ± 27.5		−14.4 ± 27.8	
Joint pain	Placebo	−8.2 ± 33.0	0.719	−13.2 ± 27.8	0.753	−18.6 ± 23.5	0.653	−17.0 ± 23.8	0.723
	RJ	−11.7 ± 29.0		−16.0 ± 28.7		−15.3 ± 22.8		−14.0 ± 29.6	
Cold hands and feet	Placebo	−9.2 ± 19.6	0.229	−5.5 ± 18.7	0.255	−4.6 ± 26.9	0.247	−3.3 ± 27.9	0.697
	RJ	−19.6 ± 33.4		−14.6 ± 30.9		−14.3 ± 26.6		0.3 ± 31.1	
Numbness in the legs or arms	Placebo	−6.4 ± 27.9	0.799	−5.7 ± 31.8	0.937	0.1 ± 38.1	0.209	−4.0 ± 35.7	0.574
	RJ	−8.9 ± 34.3		−6.5 ± 34.5		−13.3 ± 29.3		−10.0 ± 33.5	
Sensitive to sounds	Placebo	−4.2 ± 18.6	0.604	−12.6 ± 24.6	0.219	−13.5 ± 17.3	0.098	−11.4 ± 26.7	0.518
	RJ	−0.4 ± 27.7		−3.3 ± 23.6		−0.7 ± 30.0		−6.5 ± 22.4	

Menopausal symptoms scores were assessed monthly with menopausal symptoms questionnaire of Japanese women (Visual Analog Scale); RJ, enzyme-treated Royal Jelly. All values are mean ± SD.
